# Long-term efficacy and safety of direct oral anticoagulants in venous thrombotic antiphospholipid syndrome patients not candidate to warfarin: A pilot prospective case series study

**DOI:** 10.1007/s11239-025-03158-3

**Published:** 2025-08-29

**Authors:** Daniele Pastori, Danilo Menichelli, Gian Marco Podda, Bianca Clerici, Simone Birocchi, Tommaso Bucci, Paul RJ Ames, Pasquale Pignatelli

**Affiliations:** 1 Department of Clinical, Internal, Anesthesiologic and Cardiovascular Sciences, Sapienza University of Rome, Viale del Policlinico 155, Rome, 00161 Italy; 2 Department of General Surgery and Surgical Specialty Paride Stefanini, Sapienza University of Rome, Rome, 00161 Italy; 3 Struttura Complessa di Medicina Generale II, Ospedale San Paolo, ASST Santi Paolo e Carlo, Università degli Studi di Milano, Milano, Italy; 4 Liverpool Centre for Cardiovascular Science, University of Liverpool, John Moores University and Liverpool and Heart and Chest Hospital, Liverpool, UK; 5 Department of Haematology, Dumfries Royal Infirmary, Immune Response & Vascular Disease, iNOVA, 4Health, Nova Medical School, Nova University Lisbon, Lisbon, Portugal, Cargenbridge, Dumfries, UK

**Keywords:** Antiphospholipid syndrome, Anticoagulation, DOAC, Warfarin, TTR, Case series

## Abstract

Data on direct oral anticoagulants (DOACs) in venous thrombotic antiphospholipid antibody syndrome (APS) are controversial. This pilot study aimed to assess the safety and efficacy of DOACs in APS patients requiring oral anticoagulation for venous thromboembolism (VTE) but unsuitable for treatment with vitamin K antagonists (VKAs). We performed a prospective multi-centre case-series including APS patients with previous VTE who were receiving treatment with DOACs due to ineligibility for VKAs. Main outcomes were bleeding, arterial and recurrent venous thrombotic events and all-cause death. We included 18 patients (median age 59.6 years, 66.7% women). The antiphospholipid antibody pattern was single positivity for 33.3% patients, double positivity for 33.3%, and triple positivity for 27.8%. Only one patient had seronegative APS. Apixaban, dabigatran, rivaroxaban and edoxaban were prescribed in 44.4%, 27.8%, 16.7% and 11.1% of patients, respectively. The mean follow-up was 50.1 ± 24.1 months. During the observation period, no recurrent VTE episodes or arterial thrombotic events were recorded. Four bleedings, of which 2 major, were reported. The incidence rate of bleeding was 5.3 per 100 patient-years (95% confidence interval [95%CI] 1.4–13.6). No intracranial bleedings were recorded.

In conclusion, our preliminary findings may suggest DOAC as possible option for patients with venous thrombotic APS unsuitable to VKAs. Although these findings are promising, larger cohort studies are needed to confirm this finding.

## Graphical abstract

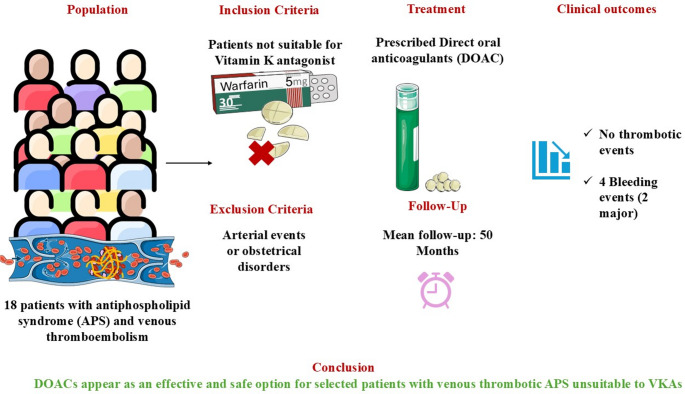

## Introduction

Patients with antiphospholipid antibody syndrome (APS) have an increased risk of venous (VTE) and arterial thromboembolism (ATE); treatment of thrombotic events and prevention of recurrences is mainly based on the administration of oral anticoagulation therapy [1, 2]. Vitamin K antagonists (VKAs), such as warfarin, have long been the standard treatment option for APS patients requiring anticoagulation. However, some patients may experience recurrences of thrombosis despite oral anticoagulation therapy [3], and high-intensity VKA therapy was proven ineffective for the prevention of recurrent VTE [4].

In addition, anticoagulation in APS patients may be challenging for a number of reasons, including the so-called warfarin resistance, the instability of anticoagulation (i.e., low time in therapeutic range, TTR) [5] and the need for frequent INR checks for VKA monitoring and dosing. Furthermore, a significant proportion of warfarin patients discontinue anticoagulation therapy during follow-up [5].

For a long time, the only alternative to VKAs was represented by aspirin, which, however, has uncertain efficacy in preventing VTE [6].

Direct oral anticoagulants (DOACs) are effective and generally safe for managing conditions such as VTE or atrial fibrillation (AF), and they represent an alternative for patients who are unsuitable for VKA therapy [7]. Of note, in patients unsuitable for VKAs, DOACs reduced the risk of thromboembolism without a significant increase of the risk of major bleeding or intracranial haemorrhage compared to aspirin.

Despite the potential advantages of DOACs, their use in patients with APS is controversial particularly following the results of the TRAPS trial, which was stopped for an excess of thrombotic and bleeding events [8]. Nevertheless, this trial’s results were limited to a specific patient subset (triple-positive APS) and included around 20% of patients with a history of arterial events, leaving uncertainty regarding the broader population of APS patients with venous thrombotic events [9].

Currently, there is no solid evidence regarding the safety and efficacy of DOACs in APS patients with single or double antiphospholipid antibodies (aPL) positivity and/or venous thrombotic events only. Current EULAR [9] and British Society Haematology guidelines suggest the use of DOACs as an alternative treatment to VKAs after a shared decision with patients [9]. This study seeks to address this gap by investigating the long-term safety and efficacy of DOACs in APS patients with VTE who not suitable candidates for VKAs are.

## Methods

We conducted a prospective, multi-centre case series including patients with APS and previous VTEs (venous thrombotic APS) treated with DOACs because they were unsuitable for VKA therapy. Patients were followed up at the anticoagulation outpatient clinics of the Policlinico Umberto I of Rome and ASST Santi Paolo e Carlo of Milan. The study included patients who fulfilled all the clinical and laboratory diagnostic APS Sydney criteria [10] and had a history of VTE but no arterial thrombotic or obstetric manifestations of APS. Two consecutive positive aPL tests, conducted at least 12 weeks apart, were required for all APS patients. Additionally, aPL levels were reassessed on a yearly basis. Before prescribing DOACs, patients were informed on current data from the literature and on the potential risks and benefits of DOACs for the management of their condition. A signed informed consent was obtained from all patients for clinical data collection. Patients with APS and ATE and/or obstetrical manifestations were excluded.

Comorbidities, concomitant therapy, laboratory tests and, for patients taking VKA before starting DOACs, TTR (using the linear interpolation method of Rosendaal [11]) were collected at baseline.

The study was approved by the Ethical Committee of Sapienza University of Rome (n° 823/17) and was performed according to the ethical guidelines of the 1975 Helsinki Declaration.

### Follow-up

After starting DOACs, patients were closely monitored with sequential fibrinogen and D-dimer testing to detect any symptomatic or asymptomatic clotting activation. Patients were regularly seen at the anticoagulation outpatient clinic for the follow-up of DOAC therapy. Patients were treated with long-term anticoagulation due to the permanent risk factor for VTE represented by APS. Records about hospital admissions and complications related to oral anticoagulation therapy (such as bleeding events) were collected during each visit. Bleeding events were classified according to the International Society on Thrombosis and Haemostasis (ISTH) classification [12]. If patients missed 1 or more visits, follow-up was performed by telephone.

### Data synthesis

Categorical variables are reported as counts (percentages). Continuous variables are expressed as means with standard deviations or medians with interquartile ranges (IQR), as appropriate. The annual incidence of adverse events was reported as the number of events per 100 patient-years, with 95% confidence intervals, calculated by dividing the total number of events by the total patient follow-up time (in years).

## Results

We included 18 patients, 12 of which were female (66.7%). Non-aggregate aPL profile and patient characteristics are reported in Table 1 (description of Methods were reported in the supplementary material). In all patients the clinical indication to oral anticoagulation therapy was VTE, but 3 patients (16.7%) had also concomitant AF.

**Table 1:** Non-aggregate clinical characteristics of eligible patients with APS and venous thromboembolism

Patient #	Sex	Age (years)	Auto-antibody positivity	Comorbidities	Venous thrombosis site (at diagnosis)
1	Female	58	LAC+	Hypertension, Parkinson’s disease	DVT of gastrocnemius and popliteal veins and SVT of lower limb
2	Male	32	LAC+	None	Monolateral PE
3	Male	77	LAC+	Hypertension, atrial fibrillation. Parkinson’s disease	DVT of lower limb
4	Female	74	LAC + aCL + aB2GPI+	Hypertension	Bilateral PE
5	Female	57	LAC + aCL + aB2GPI+	Smoking	DVT of posterior tibial vein
6	Female	57	LAC+	Hypertension, thrombocytopenia	Bilateral DVT of lower limb
7	Female	21	LAC+, aCL+	None	Bilateral PE
8	Male	63	aCL+, aB2GPI+	Depression	Recurrent DVT of lower limbs
9	Male	30	aCL/ antiVimentin +	Smoking, thrombocytopenia	PE with Pulmonary infarction
10	Female	52	LAC + aCL + aB2GPI+	None	DVT of gastrocnemius and popliteal veins
11	Male	39	LAC + aCL + aB2GPI+	Hypertriglyceridemia	DVT of cava, femoral, iliac and popliteal veins
12	Female	34	LAC+	None	DVT and SVT of lower limb
13	Female	54	LAC + aCL + aB2GPI+	Hypertension, autoimmune thyroiditis with hypothyroidism	Bilateral PE and DVT of femoral and popliteal veins
14	Female	61	LAC + aB2GPI+	SLE and rheumatoid arthtritis	Bilateral PE and DVT of popliteal and gastrocnemius veins
15	Female	91	aCL + aB2GPI+	Dementia	Bilateral PE
16	Female	88	aCL + aB2GPI+	Hypertension, Atrial fibrillation, rheumatoid arthtritis	Bilateral PE and DVT of popliteal vein and SVT of lower limb
17	Female	61	LAC + aB2GPI+	Hypertension, Atrial fibrillation, smoking, autoimmune thyroiditis with hypothyroidism	Bilateral PE
18	Male	80	LAC+	Smoking	Bilateral PE and DVT of popliteal and calf veins

Abbreviations – DVT: deep vein thrombosis, LAC: lupus anticoagulant; aB2GPI: anti-β_2_-Glycoprotein I antibody; aCL anticardiolipin antibody, PE: pulmonary embolism, SLE: systemic lupus erythematosus, SVT: superficial vein thrombosis

Aggregate laboratory values and eligible patients’ clinical characteristics are reported in Table 2. Patients’ median age was 59.6 years (IQR, 38–76). Six out of 18 patients (33.3%) were only lupus anticoagulant (LAC)-positive, 6 (33.3%) had double aPL positivity, 5 (27.8%) had triple aPL positivity, and 1 patient was affected by seronegative APS with anti-cardiolipin/vimentin positivity. None of the patients had moderate-severe kidney disease or a history of chronic liver disease. Mean haemoglobin and platelet count were 13.2 ± 1.67 g/dl and 269.9 ± 140.5 × 10^3^/µL, respectively.

**Table 2:** Aggregate characteristics of eligible patients with APS and venous thromboembolism

Variable	Values
**Clinical characteristics**	
*Age (years) – Median and IQR*	59.6 (38.0-76.2)
*Females (%)*	66.7
*Hypertension (%)*	38.9
*Atrial fibrillation (%)*	16.7
*Autoimmune diseases (%)*	22.2
*Neurological disorders (%)*	16.7
Thrombocytopenia* (%)	11.1
*Smoking (%)*	16.7
**Laboratory test**	
*Haemoglobin – Mean and SD*	13.2 ± 1.67 g/dl
*Platelet count – Mean and SD*	269.9 ± 140.5 × 10^3^/µL
*Platelet count – Mean and SD (excluding patients with thrombocytopenia)*	286.7 ± 141.35 × 10^3^/µL
*Creatinine – Mean and SD*	0.95 ± 0.31 mg/dl
*AST – Median and IQR*	19.5 (17.8–23.3)
*ALT – Median and IQR*	17.5 (11.8–20.8)
*aPL positivity profile (%)*	
*Single positivity* *Double positivity* *Triple positivity* *Seronegative APS*	33.3 33.3 27.8 5.6
**DOAC (%)**	
*Apixaban*	44.4
*Dabigatran*	27.8
*Edoxaban*	11.1
*Rivaroxaban*	16.7
*DOAC as first line of treatment (%)*	27.8
*Previous therapy with VKA (%)*	72.2

Abbreviations – IQR: interquartile range; SD: standard deviation; aPL, antiphospholipid antibody; APS: antiphospholipid antibody syndrome; DOAC: direct oral anticoagulant; VKA: vitamin K antagonist

*mild thrombocytopenia in 2 patients 148.0 × 10^3^/µL and 139.0 × 10^3^/µL

Detailed data on anticoagulation are reported in Table 3. Five out of 18 patients were prescribed a DOAC from the start of anticoagulant treatment. For the remaining 13 patients, the main reasons for switching from VKA/low-molecular-weight heparin (LMWH) to a DOAC were: (1) instability of anticoagulation with VKAs (i.e., TTR < 60%), (2) patients’ unwillingness of undergoing INR monitoring, and (3) recurrent VTE while on VKA/therapeutic LMWH dose. In detail, the reasons for switching were poor TTR in 6 patients (range 19–55%), unwillingness to continue INR monitoring in 5 patients, and recurrent VTE under VKA in 1 patient. Dabigatran was prescribed to 5 patients (27.8%; full dosage in 4 and reduced dosage in 1), apixaban to 8 patients (44.4%), 5 of which were started on apixaban 2.5 mg BID for extended thromboprophylaxis. Rivaroxaban was prescribed to 16.7% of patients (20 mg OD in 2 patients and 10 mg OD in 1 patient). Finally, low dose edoxaban was prescribed to 2 patients (11.1%); no patients received the full dose in our cohort. We monitored patients for adverse outcomes including bleeding, thrombotic events and death. The mean follow-up was 50.1 ± 24.1 months. During the observation period, no VTE relapses or other thrombotic events were recorded. All patients had persistently within normal range D-dimer values during follow-up except for Patient 12, in whom apixaban was increased from 2.5 mg BID to 5 mg BID for an asymptomatic increase of the D-dimer, which returned to normal afterwards. Patient 11, who was initially switched to a DOAC for poor TTR, returned to a VKA for no evidence of thrombus resolution under rivaroxaban. The incidence rate of bleeding in our cohort was 5.3 per 100 patient-years (95% confidence interval [95%CI] 1.4–13.6).

**Table 3:** Data on anticoagulation, reasons for switching to a DOAC and follow-up data

Patient #	DOAC type and dose	Prior oral anticoagulation therapy	Reason for switching to a DOAC	Adverse events*	Time on VKA before starting DOAC (months)
1	Dabigatran 150 mg BID	VKA	Recurrent VTE under VKA	None	6
2	Dabigatran 150 mg BID	VKA	Poor TTR (55%)	None	36
3	Dabigatran 150 mg BID	VKA	Poor TTR (39%)	No thrombotic events. minor Hemorrhoidal bleeding. Died for colorectal cancer	12
4	Dabigatran 150 mg BID	VKA	Unwilling to continue INR monitoring despite good TTR (75%) due to living alone	None	42
5	Apixaban 2.5 mg BID	VKA	Unwilling to continue INR monitoring despite good TTR (81%) for reduced mobility	None	48
6	Apixaban 5 mg BID	VKA	Unwilling to undergo frequent blood sample collection	None	42
7	Rivaroxaban 20 mg OD	VKA	Unwilling to undergo frequent blood sample collection	None	6
8	Apixaban 2.5 mg BID	VKA	Poor TTR (19%)	None	48
9	Dabigatran 110 mg BID	None	Unwilling to undergo frequent blood sample collection	None	0
10	Apixaban 2.5 mg BID	VKA	Poor TTR (32%)	None	6
11	Rivaroxaban 20 mg OD	VKA	Poor TTR (36%)	Return to VKA with good TTR (91%) due to non-resolution of VTE on rivaroxaban	12
12	Apixaban 2.5 mg BID	VKA	Poor TTR (56%)	Apixaban increased to 5 mg BID for asymptomatic elevation of the D-dimer	44
13	Rivaroxaban 10 mg OD	VKA	Renal angiomyolipoma hematoma	None	86
14	Apixaban 5 mg BID	VKA	Poor TTR, patient’s choice	None	41
15	Edoxaban 30 mg OD	None	Patient’s choice	No thrombotic events. Minor hemorrhoidal bleeding.	0
16	Edoxaban 30 mg OD	None	Patient’s choice	No thrombotic events. Major bleeding from the superior mesenteric artery during acute pancreatitis.	0
17	Apixaban 5 mg BID	None	Patient’s choice	None	0
18	Apixaban 2.5 mg BID	None	Patient’s choice	No thrombotic events. Major genitourinary bleeding.	0

Abbreviations – DOAC: direct oral anticoagulant; BID: bis in die; VKA: vitamin K antagonist; VTE, venous thromboembolism; TTR, time in therapeutic range; OD: once daily

*including bleeding events, thrombotic events and death

Of the total of 4 bleeding events occurred during follow-up, two were major bleeding events. Patient 16 experienced major bleeding from the superior mesenteric artery during acute pancreatitis while on treatment with edoxaban 30 mg OD, while patient 18 experienced major genitourinary bleeding on apixaban 2.5 BID. We recorded two minor hemorrhoidal bleedings in patients 3 and 15 (on dabigatran 150 mg BID and on edoxaban 30 mg OD, respectively). Patient 3 was eventually diagnosed with a fatal colon cancer. None of the patients died because of APS. Indeed, all the remaining patients are on active thromboprophylaxis with no other complications. No other side effects were registered. All APS patients that experienced bleeding during follow-up had no hepatic and kidney impairment and were not treated with concomitant antiplatelet therapy.

## Discussion

Our case series of 18 patients with venous thrombotic APS ineligible to VKAs, proved that DOACs are safe and effective: there were no VTE recurrences nor ATEs. During the mean follow-up of 50.1 months, none of the patients died because of APS-related causes or experienced recurrent VTE or ATE, highlighting the long-term safety and efficacy of DOACs in this specific patient population.

Our results are in line with an analysis from the RE-COVER/RE-COVER II and RE-MEDY trials, which showed a similar safety and efficacy of dabigatran compared to warfarin in patients with VTE and thrombophilia, 20% of whom had APS [9]. However, the safety of DOACs in APS patients remains a matter of debate, especially following the TRAPS trial. two metanalyses showed that the excess of thrombotic risk in APS patients treated with DOACs is due to arterial thrombosis rather than to VTE [13, 14]; however, the length of follow-up of included studies was relatively short (< 2 years) [13, 14]. Our patients were followed for a mean of 4 years, providing useful information on long-term outcomes of APS patients on DOACs for VTE. Our results are coherent with a recent observational study [15] performed on 152 APS patients (66 treated with apixaban) during a median follow-up of 53 months, that showed no difference for recurrence of VTE and major bleeding between apixaban and VKA groups [15].

The clinical management of these patients was at the discretion of the treating physicians and the choice of anticoagulation with DOACs was based on some clinical considerations, as well as on patients’ preferences. Firstly, based on guidelines and on existing literature, the use of DOACs was discussed only with patients with VTE as an indication to oral anticoagulation therapy, while patients with previous ATE were kept on warfarin. In addition, given the high prothrombotic burden of APS and the lack of data on extended anticoagulation in these patients, we decided for most patients to use full dose anticoagulation also beyond the first 6 months after the VTE event. In two cases (patients 5 and 8) we decided to adopt an extended treatment with a low-dose of apixaban after at least 6-months of treatment with VKA according to the extended management suggested by international guidelines [16, 17] and AMPLIFY-EXT trial [18], taking account also the type of venous thrombosis (distal deep vein thrombosis for patient 5) and aPL profile (low-risk profile for patient 8).

The use of low dose extended therapy as apixaban 2.5 mg BID [18] or rivaroxaban 10 mg OD [19] was investigated in two clinical trials and showed a low recurrence of VTE compared to placebo. However, no direct comparison between full and low dose of DOAC was available in VTE patients, especially ones with APS. Recently a small, randomized trial, the ASTRO-APS [20], investigated the use of apixaban 2.5 mg BID compared to VKA in APS patients. Apixaban group had a higher number of ischemic stroke events, while no VTE were registered in both groups. However, the trial was stopped prematurely, and the small sample size did not give strong evidence.

Finally, when possible, we preferred DOACs with double daily administration to minimize the time in which patients are exposed to low through plasma levels.

Moreover, the careful selection of patients was accompanied by close monitoring of D-dimer levels during follow-up. This may partially explain the low rate of thrombotic events we found in our study.

Although the DULCIS trial [21] evaluated the role of D-dimer during follow-up to establish patients with low-risk of VTE recurrence and anticoagulation duration, no data was available on the use of D-dimer for the choice of anticoagulant dose. Furthermore, an asymptomatic rise of D-dimer did not reflect the presence of venous thrombosis, so the clinical choice to increase apixaban dose in patient 12 is still a matter of debate and was not supported by clinical evidence.

During follow up, 4 bleeding events occurred of which 2 were major. Although our data were limited by small sample size, the incidence of 5.3 hemorrhages per 100 patient-year found in our case series is similar to that reported in previous clinical trials on DOAC in patients with VTE.

The results of our study should be read in light of its limitations. Despite the fact that data were collected within a multi-center study, we have a limited sample size underpowered to detect differences in thrombotic and bleeding events with heterogeneous patients, as most patients had single-positive or double-positive APS (66.6%), whereas the number of triple-positive patients was low. In particular, there were no events in triple-positive patients of our cohort, but the low sample did not permit to ensure the effectiveness and safety of DOACs in these patients.

Moreover, no comparisons were made with similar patients prescribed VKA, limiting the strength of the evidence derived regarding DOACs efficacy and safety. The majority of our study population (72.2%) was on treatment with DOACs administered twice daily, thus our findings mainly apply to dabigatran and apixaban. Additionally, the absence of thrombotic events, although encouraging, was likely overestimated due to the low sample size and the exclusion of patients with previous arterial thrombotic events or triple positivity, in which warfarin remains the standard of care [2]. Finally, the majority of patients were enrolled due to unsuitability for VKA, and this may represent a potential selection bias.

In conclusion, currently the use of DOACs should be cautious due to controversies raised by international guidelines [9]. Preliminary findings from this prospective series highlight the potential role of DOACs in venous thrombotic APS patients who are ineligible for VKA therapy. Nevertheless, given the limited sample size and the underrepresentation of high-risk subgroups, such as triple-positive patients, larger studies are necessary to confirm these findings and explore the long-term safety of DOACs across diverse APS profiles.

## Data Availability

Data that support the findings of this study are available from the corresponding author upon reasonable request.

## References

[1] Lim W, Crowther MA, Eikelboom JW (2006) Management of antiphospholipid antibody syndrome: a systematic review. JAMA: J Am Med Association 295(9):1050–1057. 10.1001/jama.295.9.1050 10.1001/jama.295.9.1050 16507806

[2] Cohen H, Werring DJ, Chandratheva A, Mittal P, Devreese KMJ, Isenberg DA et al (2023) Survey on antiphospholipid syndrome diagnosis and antithrombotic treatment in patients with ischemic stroke, other brain ischemic injury, or arterial thromboembolism in other sites: communication from ISTH SSC subcommittee on lupus anticoagulant/antiphospholipid antibodies. J Thromb Haemost 21(10):2963–2976. 10.1016/j.jtha.2023.06.020 37391096 10.1016/j.jtha.2023.06.020

[3] Radin M, Schreiber K, Cecchi I, Roccatello D, Cuadrado MJ, Sciascia S (2018) The risk of ischaemic stroke in primary antiphospholipid syndrome patients: a prospective study. Eur J Neurol 25(2):320–325. 10.1111/ene.13499 29082583 10.1111/ene.13499

[4] Finazzi G, Marchioli R, Brancaccio V, Schinco P, Wisloff F, Musial J et al (2005) A randomized clinical trial of high-intensity warfarin vs. conventional antithrombotic therapy for the prevention of recurrent thrombosis in patients with the antiphospholipid syndrome (WAPS). J Thromb Haemostasis: JTH 3(5):848–853. 10.1111/j.1538-7836.2005.01340.x 15869575 10.1111/j.1538-7836.2005.01340.x

[5] Pastori D, Parrotto S, Vicario T, Saliola M, Mezzaroma I, Violi F et al (2016) Antiphospholipid syndrome and anticoagulation quality: a clinical challenge. Atherosclerosis 244:48–50. 10.1016/j.atherosclerosis.2015.10.105 26584138 10.1016/j.atherosclerosis.2015.10.105

[6] Arnaud L, Mathian A, Ruffatti A, Erkan D, Tektonidou M, Cervera R et al (2014) Efficacy of aspirin for the primary prevention of thrombosis in patients with antiphospholipid antibodies: an international and collaborative meta-analysis. Autoimmun Rev 13(3):281–291. 10.1016/j.autrev.2013.10.014 24189281 10.1016/j.autrev.2013.10.014

[7] Menichelli D, Del Sole F, Di Rocco A, Farcomeni A, Vestri A, Violi F et al (2021) Real-world safety and efficacy of direct oral anticoagulants in atrial fibrillation: a systematic review and meta-analysis of 605 771 patients. Eur Heart J Cardiovasc Pharmacother 7(FI1):f11–f9. 10.1093/ehjcvp/pvab002 33493255 10.1093/ehjcvp/pvab002

[8] Pengo V, Denas G, Zoppellaro G, Jose SP, Hoxha A, Ruffatti A et al (2018) Rivaroxaban vs warfarin in high-risk patients with antiphospholipid syndrome. Blood 132(13):1365–1371. 10.1182/blood-2018-04-848333 30002145 10.1182/blood-2018-04-848333

[9] Pastori D, Menichelli D, Cammisotto V, Pignatelli P (2021) Use of direct oral anticoagulants in patients with antiphospholipid syndrome: A systematic review and comparison of the international guidelines. Front Cardiovasc Med 8:715878. 10.3389/fcvm.2021.715878 34414220 10.3389/fcvm.2021.715878 PMC8368436

[10] Miyakis S, Lockshin MD, Atsumi T, Branch DW, Brey RL, Cervera R et al (2006) International consensus statement on an update of the classification criteria for definite antiphospholipid syndrome (APS). J Thromb Haemost 4(2):295–306. 10.1111/j.1538-7836.2006.01753.x 16420554 10.1111/j.1538-7836.2006.01753.x

[11] Rosendaal FR, Cannegieter SC, van der Meer FJ, Briet E (1993) A method to determine the optimal intensity of oral anticoagulant therapy. Thromb Haemost 69(3):236–239 8470047

[12] Pastori D, Lip GYH, Farcomeni A, Del Sole F, Sciacqua A, Perticone F et al (2018) Data on incidence of bleeding in patients with atrial fibrillation and advanced liver fibrosis on treatment with vitamin K or non-vitamin K antagonist oral anticoagulants. Data Brief 17:830–836. 10.1016/j.dib.2018.01.109 29527545 10.1016/j.dib.2018.01.109 PMC5842291

[13] Lee YH, Song GG (2022) Direct oral anticoagulants versus warfarin in patients with antiphospholipid syndrome: A meta-analysis of randomized controlled trials. Lupus 31(11):1335–1343. 10.1177/09612033221118463 35968627 10.1177/09612033221118463

[14] Khairani CD, Bejjani A, Piazza G, Jimenez D, Monreal M, Chatterjee S et al (2023) Direct oral anticoagulants vs vitamin K antagonists in patients with antiphospholipid syndromes: Meta-Analysis of randomized trials. J Am Coll Cardiol 81(1):16–30. 10.1016/j.jacc.2022.10.008 36328154 10.1016/j.jacc.2022.10.008 PMC9812926

[15] Sikorska M, Chmiel J, Papuga-Szela E, Broniatowska E, Undas A (2024) Apixaban versus vitamin K antagonists in patients with antiphospholipid syndrome: A cohort study. J Cardiovasc Pharmacol 84(1):36–44. 10.1097/FJC.0000000000001578 38922590 10.1097/FJC.0000000000001578

[16] Konstantinides SV, Meyer G, Becattini C, Bueno H, Geersing GJ, Harjola VP et al (2020) 2019 ESC guidelines for the diagnosis and management of acute pulmonary embolism developed in collaboration with the European respiratory society (ERS). Eur Heart J 41(4):543–603. 10.1093/eurheartj/ehz405 31504429 10.1093/eurheartj/ehz405

[17] Ortel TL, Neumann I, Ageno W, Beyth R, Clark NP, Cuker A et al (2020) American society of hematology 2020 guidelines for management of venous thromboembolism: treatment of deep vein thrombosis and pulmonary embolism. Blood Adv 4(19):4693–4738. 10.1182/bloodadvances.2020001830 33007077 10.1182/bloodadvances.2020001830 PMC7556153

[18] Agnelli G, Buller HR, Cohen A, Curto M, Gallus AS, Johnson M et al (2013) Apixaban for extended treatment of venous thromboembolism. N Engl J Med 368(8):699–708. 10.1056/NEJMoa1207541 23216615 10.1056/NEJMoa1207541

[19] Weitz JI, Lensing AWA, Prins MH, Bauersachs R, Beyer-Westendorf J, Bounameaux H et al (2017) Rivaroxaban or aspirin for extended treatment of venous thromboembolism. N Engl J Med 376(13):1211–1222. 10.1056/NEJMoa1700518 28316279 10.1056/NEJMoa1700518

[20] Woller SC, Stevens SM, Kaplan D, Wang TF, Branch DW, Groat D et al (2022) Apixaban compared with warfarin to prevent thrombosis in thrombotic antiphospholipid syndrome: a randomized trial. Blood Adv 6(6):1661–1670. 10.1182/bloodadvances.2021005808 34662890 10.1182/bloodadvances.2021005808 PMC8941474

[21] Palareti G, Cosmi B, Legnani C, Antonucci E, De Micheli V, Ghirarduzzi A et al (2014) D-dimer to guide the duration of anticoagulation in patients with venous thromboembolism: a management study. Blood 124(2):196–203. 10.1182/blood-2014-01-548065 24879813 10.1182/blood-2014-01-548065

